# The Ubiquitin C-Terminal Hydrolase L1 (UCH-L1) C Terminus Plays a Key Role in Protein Stability, but Its Farnesylation Is Not Required for Membrane Association in Primary Neurons*

**DOI:** 10.1074/jbc.M114.557124

**Published:** 2014-10-17

**Authors:** Paul Bishop, Philip Rubin, Andrew R. Thomson, Dan Rocca, Jeremy M. Henley

**Affiliations:** From the School of Biochemistry, Medical Sciences Building, University of Bristol, Bristol BS8 1TD, United Kingdom

**Keywords:** Membrane Protein, Neurobiology, Posttranslational Modification (PTM), Protein Misfolding, Ubiquitination

## Abstract

Ubiquitin C-terminal hydrolase L1 (UCH-L1) is a deubiquitinating enzyme that is highly expressed in neurons. A possible role for UCH-L1 in neurodegeneration has been highlighted because of its presence in Lewy bodies associated with Parkinson disease and neurofibrillary tangles observed in Alzheimer disease. UCH-L1 exists in two forms in neurons, a soluble cytoplasmic form (UCH-L1^C^) and a membrane-associated form (UCH-L1^M^). Alzheimer brains show reduced levels of soluble UCH-L1^C^ correlating with the formation of UCH-L1-immunoreactive tau tangles, whereas UCH-L1^M^ has been implicated in α-synuclein dysfunction. Given these reports of divergent roles, we investigated the properties of UCH-L1 membrane association. Surprisingly, our results indicate that UCH-L1 does not partition to the membrane in the cultured cell lines we tested. Furthermore, in primary cultured neurons, a proportion of UCH-L1^M^ does partition to the membrane, but, contrary to a previous report, this does not require farnesylation. Deletion of the four C-terminal residues caused the loss of protein solubility, abrogation of substrate binding, increased cell death, and an abnormal intracellular distribution, consistent with protein dysfunction and aggregation. These data indicate that UCH-L1 is differently processed in neurons compared with clonal cell lines and that farnesylation does not account for the membrane association in neurons.

## Teaser

**Background:** Membrane-associated UCH-L1 has been proposed as a target for treating neurodegeneration.

**Results:** UCH-L1 association with neuronal membranes does not involve farnesylation. C-terminal truncation causes protein misfolding and neuronal death.

**Conclusion:** The C terminus of UCH-L1 regulates protein aggregation and stability.

**Significance:** The CTTΔ4 mutant provides a model for studying neurotoxic UCH-L1 aggregation events.

## Introduction

Ubiquitination is a dynamic, posttranslational modification that is fundamentally important in controlling multiple aspects of neuronal function, including turnover, trafficking, and synaptic plasticity (1, 2). Indeed, dysfunction of ubiquitin metabolism is a hallmark of many neurodegenerative diseases, and abnormal accumulation of ubiquitinated proteins is associated with Parkinson disease, Alzheimer disease, and amyotrophic lateral sclerosis (3, 4).

Ubiquitin C-terminal hydrolase L1 (UCH-L1) is a highly abundant neuronal deubiquitinase, a cysteine protease that cleaves small peptide adducts from the C terminus of ubiquitin. Despite the fact that UCH-L1 comprises 1–5% of total neuronal protein (5) and has been shown to play a role in monoubiquitin homeostasis (6), the wider functional roles of UCH-L1 *in vivo* remain to be determined.

UCH-L1 binds ubiquitin with high affinity but possesses poor hydrolase activity *in vitro*, especially in comparison with the more widely expressed UCH-L3, with which it shares 52% sequence homology (7). Aside from its function as a deubiquitinase, it has been suggested that UCH-L1 can dimerize to act as an E4 ubiquitin ligase, resulting in accumulation of polyubiquitinated α-synuclein (8).

Interestingly, UCH-L1 can partition into both cytosolic (UCH-L1^C^) and membrane (UCH-L1^M^) fractions. In neurons, it has been estimated that 20–50% is membrane-associated (9–11), but the mechanisms and consequences of neuronal membrane association are not understood.

α-Synuclein accumulates in the insoluble protein aggregates characteristic of Parkinson disease and other synucleinopathies (12). Intriguingly, previous studies have suggested that that membrane-associated UCH-L1 can stabilize α-synuclein levels (8, 9) and that these effects vary under normal or pathological conditions (13), raising the possibility that UCH-L1^M^ could be a target for neurodegenerative drug therapy (9).

One report has suggested that the membrane association of UCH-L1 is due to farnesylation of an atypical consensus site (9). Therefore, given the potentially important implications for progression and treatment of neurodegenerative disease, we sought to further characterize the membrane-associated form of UCH-L1.

In this study we did not detect significant levels of membrane-associated UCH-L1 in any of the clonal cell lines we investigated, suggesting that membrane-association of UCH-L1 is neuron-specific. Our results also indicate that the membrane association of UCH-L1 in neurons is not dependent on farnesylation or interaction with ubiquitin or species containing ubiquitin-like folds because the C220S, C90S, and D30K mutations did not reduce the proportion of UCH-L1 in the membrane fraction. Furthermore, deletion of the final four amino acids of UCH-L1 (CKAA) caused the loss of ubiquitin substrate binding, increased neuronal death, altered UCH-L1 distribution within neurons, and removed soluble, cytoplasmic UCH-L1 with a corresponding increase in Triton X-100-insoluble UCH-L1. A proportion of truncated UCH-L1 was still detected in the membrane fraction, but the levels of soluble cytosolic UCH-L1 were significantly decreased. Circular dichroism analysis of WT and a C-terminal truncation mutant (CTTΔ4) revealed that the removal of the last four residues of UCH-L1 caused a minor disruption to the secondary structure but dramatically increased aggregation.

These changes are in line with previous reports on the disruption to the tertiary structure and aggregation of UCH-L1 and are consistent with the distribution and soluble properties of UCH-L1 in Alzheimer brains (14, 15), suggesting that this C-terminal truncation may provide a model for studying neuronal cell death arising from UCH-L1 misfolding and aggregation.

## EXPERIMENTAL PROCEDURES

### 

#### 

##### Cell Culture

Primary hippocampal and cortical neuronal cultures were prepared from embryonic day 18 rats exactly as described previously (16). The N2a, HEK293T, and COS7 cell lines were maintained using standard protocols, and N2a cell differentiation and neurite outgrowth were induced by serum starvation for 24 h.

##### Immunoblotting

All SDS-PAGE and Western blotting procedures were carried out according to standard protocols. The following primary antibodies were used: mouse anti-UCH-L1 (31A3, a gift from Professor Ian Day, University of Bristol, and Prof. Rod Thompson, 1:10,000), rabbit anti-GluA1 (Millipore, 1:1000), rabbit anti-RhoGDI (Abcam, 1:5000), mouse anti-H-ras (Santa Cruz Biotechnology, 1:1000), mouse anti-Rac1 (Santa Cruz Biotechnology, 1:1000), rabbit anti-HA (Abcam, 1:5000), goat anti-Lamin B (Santa Cruz Biotechnology, 1:2000), and rabbit anti-PSD95 (Abcam, 1:1000). All blots were developed using Li-Cor Odyssey imaging software, except for anti-Lamin B, which was developed on film using HRP-conjugated anti-goat (Sigma, 1:10,000). For Li-Cor Odyssey Western blot imaging, the IRDye secondary antibodies (all from Li-Cor) used were 800CW donkey anti-mouse, 800CW donkey anti-rabbit, 680RD donkey anti-mouse, and 680RD donkey anti-rabbit, all 1:10,000. Western blot signal quantification was performed using Li-Cor Odyssey imaging software.

##### Subcellular Fractionation of Rat Brain

Fractions were obtained as described previously (17).

##### Subcellular Fractionation of Day in Vitro 18 Cultured Cortical Neurons and Cell Lines

Cells were scraped in detergent-free subcellular fractionation buffer (250 mm sucrose, 20 mm HEPES (pH 7.4), 10 mm KCl, 1.5 mm MgCl_2_, 1 mm EDTA, 1 mm DTT, and protease inhibitors (Roche)), lysed by passing the cell suspension through a 25-gauge needle 12 times using a 1-ml syringe, and then sonicated briefly three times on low power. Lysates were centrifuged at 100,000 × *g* in a benchtop ultracentrifuge (Beckman) for 1 h at 4 °C. The supernatant was retained as the cytosolic fraction. The pellet was washed three times in subcellular fractionation buffer and resuspended in a standard lysis buffer containing 1% Triton and 0.1% SDS and solubilized on a rotating wheel at 4 °C for 30 min. The suspension was centrifuged at 10,000 × *g* in a benchtop centrifuge for 10 min at 4 °C. The supernatant was retained as the membrane fraction. The Triton X-100-insoluble fraction was prepared by resuspending the remaining pellet in lysis buffer containing 1% Triton and 0.1% SDS, rotated at 4 °C for 30 min, passed through a 25-gauge needle 12 times using a 1-ml syringe, and then sonicated three times on low power. Protein concentration was determined using a Bradford assay (Bio-Rad) to determine protein concentration compared with a set of BSA standards. Sarkosyl solubility was determined by resuspending the Triton X-100-insoluble fraction in lysis buffer containing 1% *N*-lauroylsarcosine sodium salt (Sigma), passed through a needle and sonicated as before, rotated at room temperature for 1 h, and ultracentrifuged at 100,000 × *g* at 4 °C for 45 min. 20 μl of the supernatant was loaded on the gel as the sarkosyl-soluble fraction. The remaining pellet was resuspended in lysis buffer containing 2% SDS, passed through a needle, resonicated, and then rotated on a wheel at room temperature for a further 30 min. 20 μl was loaded on the gel as the sarkosyl-insoluble fraction.

##### Farnesyltransferase Inhibitor Assay

Cultured cortical neurons were treated with DMSO (Sigma) or the farnesyltransferase inhibitor FTI-276 (Calbiochem) for 48 h prior to undergoing the subcellular fractionation protocol at day *in vitro* 18 as described above.

##### Molecular Biology

The GFP-UCH-L1 human cDNA plasmid was a gift from Prof. Mike Clague (University of Liverpool). His6-TAT-HA-UCH-L1 was a gift from Dr. Ottavio Arancio. HA-UCH-L1 constructs were generated using standard cloning procedures and PCR-based mutagenesis. Sindbis virus expressing HA-UCH-L1 constructs were created using the pSinRep5 plasmid and Ambion virus production tools.

##### UbVME Binding Assay

Cultured cortical neurons were infected with Sindbis virus-expressing HA-UCH-L1 constructs for 24 h prior to lysis. N2a cells were transfected with HA-UCH-L1 constructs at ∼75% confluency using Lipofectamine LTX Plus (Invitrogen) for 40 h prior to lysis. 20 μg of cell lysates was incubated with 2 μm of UbVME^2^ substrate (Boston Biochem) for 1 h at 37 °C and then subjected to SDS-PAGE.

##### Microscopy

For fixed cell confocal imaging, standard 4% PFA fixation and immunostaining protocols were used on day *in vitro* 18 dissociated hippocampal neurons infected with Sindbis virus expressing HA-UCH-L1 constructs for 24 h. Anti-UCH-L1 and anti-HA antibodies were used at 1:500. Fluorophore-conjugated secondary antibodies used were goat anti-mouse-DyLight 488 and goat anti-rabbit DyLight 649 (Jackson ImmunoResearch Laboratories) and Hoechst nuclear stain (Thermo Scientific, 1:10,000). Confocal imaging was performed using a ×63 oil immersion objective lens (numerical aperture, 1.4) on a Zeiss LSM 150 META inverted confocal microscope. Maximum projection confocal z stacks were taken using 0.4-μm steps using a 1024 × 1024 pixel resolution and a pinhole ∼1 airy unit and prepared using ImageJ software (National Institutes of Health).

##### Cell Death Assay

lactate dehydrogenase levels in conditioned neuronal culture media were assessed using an *in vitro* toxicology assay kit (Sigma). Briefly, day *in vitro* 18 dissociated cortical neurons cultured in 3-cm dishes were infected with Sindbis virus expressing HA-UCH-L1 constructs for 24 h. 100 μl of conditioned culture medium was taken from each dish. 30 μl of medium was transferred to each well of a 96-well plate. A lactate dehydrogenase assay mixture containing substrate, cofactor, and dye solutions was prepared according to the instructions of the manufacturer, and 60 μl of the mixture was added to each well of a 96-well plate. Following 25 min of incubation, the reaction was quenched using 10 μl of 1 N HCl, and the absorbance at 492 nm and at reference 620 nm was measured using a Tecan program on a plate reader.

##### Circular Dichroism

Purified TAT-HA-UCH-L1 and TAT-HA-CTTΔ4 were analyzed by circular dichroism spectroscopy at a concentration of 5 μm in PBS with 1 mm Tris (2-carboxyethyl)phosphine and 0.01% SDS. CD data were collected on a Jasco J810 spectropolarimeter fitted with a Peltier temperature-controlled sample stage. Samples were placed in a 1-mm path length quartz cuvette (Starna), and the scan rate was 50 nm min^−1^ with a 1-nm interval, a 1-nm bandwidth, and a 1-s response time.

## RESULTS

### 

#### 

##### UCH-L1 Partitions to the Membrane in Primary Cultured Cortical Neurons but Not in Clonal Cell Lines

**FIGURE 1.**
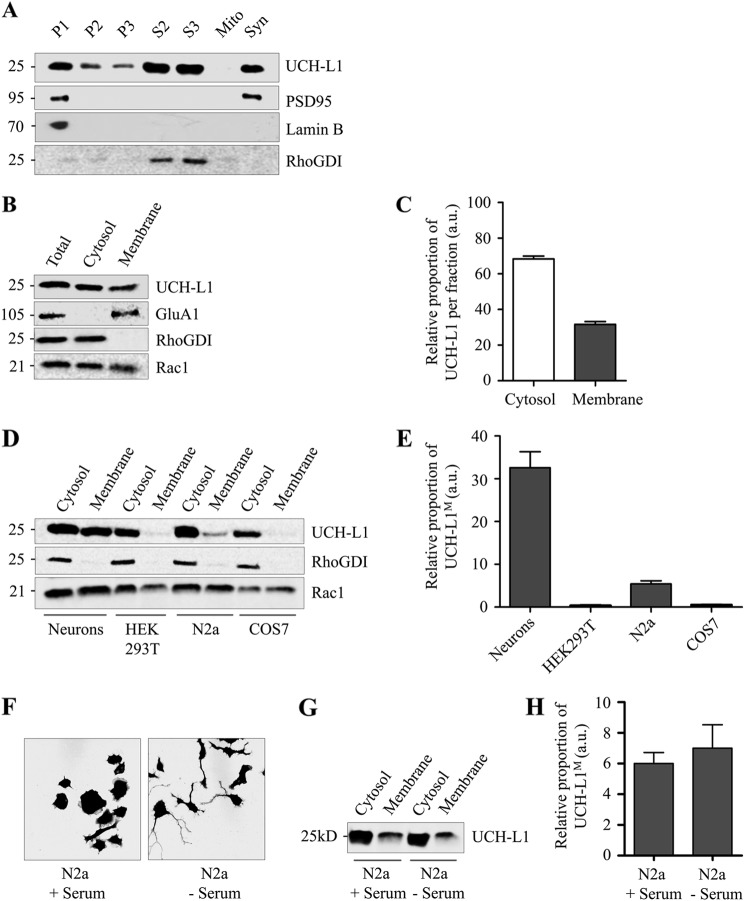
**UCH-L1 exists in both cytosolic and membrane-associated forms in neurons.** Cell lines contain much lower levels of membrane-associated UCH-L1 than neurons. *A*, UCH-L1 exists in cytosolic and membrane-enriched fractions of whole rat brain. Adult male rat brain was homogenized and subjected to subcellular fractionation. Fractions were immunoblotted for protein markers to verify enrichment. 30 μg of protein was loaded in each lane. Numbers shown are in kilodaltons relative to molecular weight standards. *P1*, nuclear-enriched fraction; *P2*, crude membranes and synaptosomes; *P3*, microsomes, including the endoplasmic reticulum and Golgi; *S2*, cytosol and microsomes; *S3*, cytosol; Mito, mitochondria; *Syn*, synaptosomes. *B*, UCH-L1 exists in cytosolic and membrane fractions of cultured cortical neurons. Rat cortical neuronal lysate was subjected to high-speed ultracentrifugation to isolate the soluble cytosolic supernatant and membrane pellet fractions. Protein distribution was tested by immunoblotting for soluble cytosolic protein (RhoGDI) and transmembrane protein (GluA1). A proportion of Rac1 in neurons is peripherally associated with the membrane via geranylgeranylation, and the endogenous distribution is preserved using this protocol. 15 μg of protein was loaded in each lane. *C*, relative proportions of UCH-L1 detected per microgram of protein in the cytosolic and membrane fractions of cultured cortical neurons. The UCH-L1 signal was quantified using Li-Cor Odyssey imaging software. *a.u.,* arbitrary units. *D*, cell lines contain much lower levels of UCH-L1^M^ than cultured cortical neurons. Lysates from the indicated clonal cell lines were subjected to high-speed ultracentrifugation to isolate the cytosolic and membrane fractions. Protein distribution was tested by immunoblotting for cytosolic protein (RhoGDI), and prenylated protein (Rac1). The distribution of Rac1 is preserved in cell lines, although the proportions vary between cell lines. UCH-L1 was not detected in the membrane fraction of COS7 or HEK293T cells and only to a minor extent with increased gain in N2a cells. 20 μg of protein was loaded in each lane. *E*, relative proportions of UCH-L1 detected per microgram of protein in the cytosolic and membrane fractions of the indicated cell lines. The UCH-L1 signal was quantified using Li-Cor Odyssey imaging software. *F*, serum starvation induces outgrowth of neuronal-like processes in N2a cells. GFP-transfected N2a cells were exposed to serum starvation for 24 h and fixed for confocal microscopy. Cell outlines were identified, and images were prepared using ImageJ software with MacBiophotonics plug-ins. *G*, differentiation does not alter the proportion of UCH-L1^M^ in N2a cells. Outgrowth of neuronal-like processes was induced in N2a cells with 24-h serum starvation, and then cells were subjected to ultracentrifugation as before. The proportion of UCH-L1^M^ remained unchanged compared with untreated N2a cells. *H*, relative proportions of UCH-L1^M^ detected per microgram of protein in the membrane fraction of N2a cells. The UCH-L1 signal was quantified using Li-Cor Odyssey imaging software.

Subcellular fractionation of adult rat brain was used to identify the neuronal compartments that contain UCH-L1. As expected, UCH-L1 was most abundant in the cytosolic fractions, but it was also detected in several of the membrane-enriched fractions (Fig. 1*A*). Immunoblotting for the cytosolic marker RhoGDI showed that the presence of UCH-L1 in these fractions was not due to cytosolic contamination.

To determine the proportion of membrane to cytosolic UCH-L1, we next optimized a subcellular fractionation protocol for cultured cortical neurons to efficiently separate soluble cytosolic proteins from transmembrane proteins, which we validated by immunoblotting for cytosolic (RhoGDI) and membrane (GluA1) protein markers (Fig. 1*B*). Importantly, we also confirmed that this protocol preserves the cytosolic/membrane ratio of proteins that partition into both compartments by analyzing Rac1. Rac1 is a partially prenylated protein that is posttranslationally modified by geranylgeranylation (18) leading to both cytosolic and peripheral membrane-associated forms (Fig. 1*B*). UCH-L1 was detected by Western blot in both the cytosolic and membrane fractions (Fig. 1*B*) with ∼30% UCH-L1^M^ (Fig. 1*C*), consistent with values reported previously and, potentially, for a role of farnesylation in regulating membrane partitioning (9, 11).

Farnesylation involves the irreversible posttranslational addition of a 15-carbon lipid tail to the cysteine at the C-terminal end of proteins containing the sequence C*AAX* (where C is a cysteine residue, *A* is any aliphatic amino acid, and *X* can be any amino acid), with the resulting cleavage of the final three residues (19). The membrane association of UCH-L1 in COS7 cells has been reported to be due to farnesylation of an atypical consensus sequence (CKAA) at its C terminus (9). However, in contrast to that report and to our own data using cultured neurons, we were unable to detect any appreciable amounts of UCH-L1^M^ in the membrane fraction of COS7 or HEK293T cells, although they expressed endogenous UCH-L1^C^. UCH-L1^M^ was detected weakly in mouse neuroblastoma-derived N2a cells (∼5.8%) compared with cultured cortical neurons (∼32.2%) (Fig. 1, *D* and *E*). To test whether the proportion of UCH-L1^M^ in neurons is linked to neurite outgrowth, we induced N2a cell differentiation. 24 h of serum starvation produced neuronal-like processes characteristic of differentiation, but the proportion of UCH-L1^M^ was unchanged (Fig. 1, *F* and *H*). Taken together, our data strongly suggest that membrane association occurs selectively in neurons but is not dependent on differentiation.

##### Inhibition of Farnesylation Does Not Alter the Localization of UCH-L1^M^

**FIGURE 2.**
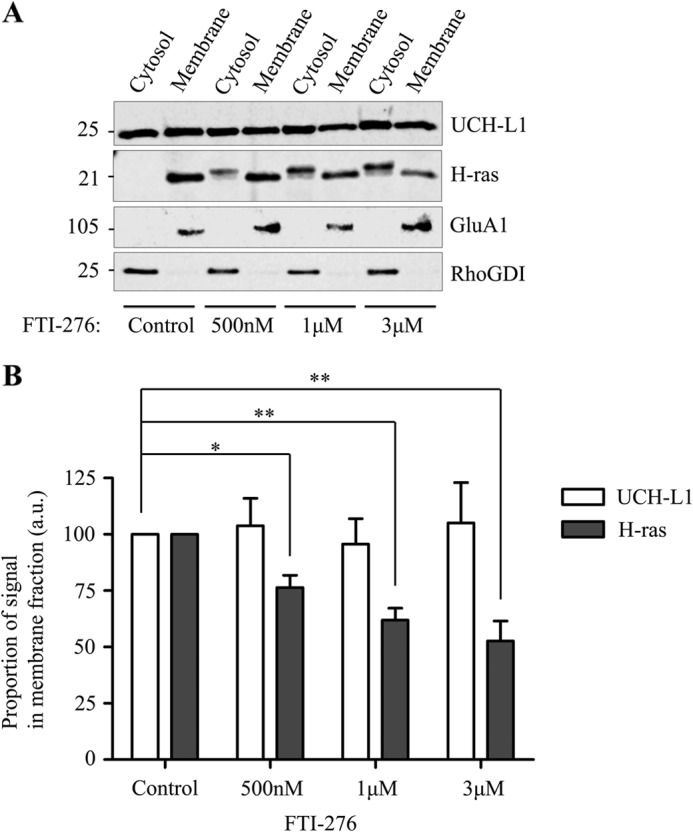
**The farnesyltransferase inhibitor FTI-276 does not reduce UCH-L1^M^ in neurons.** *A*, membrane-associated H-ras, but not UCH-L1, is decreased by farnesyltransferase inhibition. Cultured cortical neurons were treated with a DMSO control or increasing amounts of the farnesyltransferase inhibitor FTI-276 (Calbiochem), as indicated, for 48 h and then lysed and subjected to high-speed ultracentrifugation to separate the cytosolic and membrane fractions. Immunoblotting for the known farnesylation substrate H-ras shows an increasing shift from a membrane-associated form to a cytosolic form as the concentration of FTI-276 increases, whereas the proportion of UCH-L1 remains unaffected. *B*, relative proportions of UCH-L1^M^ and H-ras^M^ detected per microgram of protein in the FTI-treated membrane fractions relative to the DMSO control (*n* = 6 for the DMSO control and 500 nm, and *n* = 5 for both 1 and 3 μm). Data were analyzed using one-way analysis of variance with post hoc Bonferroni test. *, *p* < 0.05; **, *p* < 0.001. *a.u.,* arbitrary units.

Despite UCH-L1 being a predominantly neuronal protein, the effects of UCH-L1 farnesylation have not been investigated previously in neurons. We therefore tested the sensitivity of UCH-L1^M^ to the farnesyltransferase inhibitor FTI-276. Because membrane-association of H-ras is mediated by farnesylation at the C terminus (20), as a positive control we first assessed whether inhibition of farnesyltransferase disrupted localization of H-ras (21). As shown in Fig. 2, *A* and *B*, increasing concentrations of FTI-276 disrupted H-ras membrane binding and led to the appearance of H-ras in the cytosolic fractions. In stark contrast, however, FTI-276 did not alter the proportions of UCH-L1^C^ and UCH-L1^M^, indicating that membrane association of UCH-L1 in neurons is not dependent on farnesylation.

##### Membrane Association of UCH-L1 Is Not Dependent on Modification at Key Cysteine Residues or Ubiquitin Binding

**FIGURE 3.**
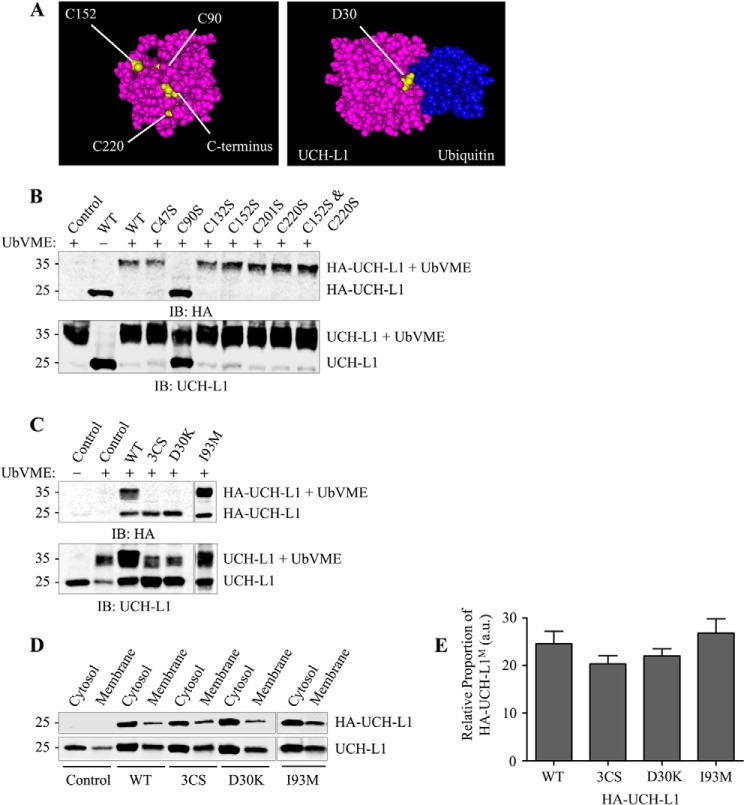
**Selected mutations do not reduce membrane-associated UCH-L1^M^.** *A*, three-dimensional models showing the location of visible mutated or deleted residues in UCH-L1 using Cn3D software with UCH-L1 PDB codes 2ETL and 3KW5 (bound to UbVME). The Ile-93 residue is buried internally. *B*, effect of individual UCH-L1 cysteine mutations on UbVME binding in N2a cells. N2a cells were transfected with HA-UCH-L1 Cys-to-Ser point mutants as indicated. Cell lysates were incubated with 2 μm UbVME to assess the effect of mutation on substrate binding and then immunoblotted (*IB*) to detect infected HA-UCH-L1 constructs and total (endogenous + infected) UCH-L1. *C*, 3CS (C90S, C152S, and C220S) and D30K mutations abolish UbVME binding, but I93M does not. Cultured cortical neurons were infected with Sindbis virus expressing the indicated HA-tagged UCH-L1 constructs. Lysates were incubated with 2 μm UbVME to assess the effect of mutations on substrate binding and then immunoblotted to detect infected HA-UCH-L1 constructs and total (endogenous and infected) UCH-L1. *D*, 3CS (C90S, C152S, and C220S), D30K, and I93M mutations do not reduce the proportion of UCH-L1^M^. Cultured cortical neurons were infected with Sindbis virus expressing the indicated HA-tagged UCH-L1. Lysates were subjected to ultracentrifugation to separate cytosolic and membrane-associated proteins. The fractions were immunoblotted to detect infected HA-UCH-L1 constructs and total (endogenous and infected) UCH-L1. *E*, relative proportions of the indicated HA-UCH-L1^M^ constructs detected per microgram of protein in the membrane fraction relative to the cytosolic fraction (WT, *n* = 9; 3CS, *n* = 3; D30K, *n* = 5; I93M, *n* = 3). *a.u.,* arbitrary units.

Several posttranslational modifications that generate membrane association, such as prenylation (farnesylation and geranylgeranylation) and palmitoylation, rely on covalent attachments to cysteine residues. We therefore mutated three cysteine residues in UCH-L1 (3CS) Cys-90, Cys-152, Cys-220) because these side chains are likely accessible for modification in the cytosol. Although Cys-90 is part of the catalytic triad in the active site, Cys-152 can bind lipid products that mediate the cellular response to oxidative stress and inflammation (15), and Cys-220 has been proposed as the site of farnesylation (9). None of the cysteine residues are predicted to form disulfide bonds, and mutation to serine was chosen over alanine to better preserve the space-filling properties of the side chains and to keep any conformational disruption to a minimum (Fig. 3*A*).

Mutation of each of the six cysteine residues in HA-tagged UCH-L1 was tested in N2a cells to assess whether UCH-L1 could still bind covalently to a ubiquitin vinyl methyl ester suicide substrate (Boston Biochem), which forms a bond with Cys-90 in the active site. Notably, the C152S and C220S mutants, individually or combined, showed comparable substrate binding compared with the WT, whereas binding was abolished in the C90S mutant (Fig. 3*B*). These data suggest that mutation of any individual cysteine residue does not disrupt UCH-L1 conformation.

We next infected cultured cortical neurons with WT or the triple cysteine mutant HA-UCH-L1 (3CS). As shown in Fig. 3*C*, HA-UCH-L1 bound to UbVME, and ∼25% partitioned into the membrane, consistent with ∼30% of endogenously expressed UCH-L1 (Fig. 3, *D* and *E*). Therefore, the HA-tagged virally overexpressed UCH-L1 behaves in a similar manner as endogenous protein. Interestingly, ∼20% of the HA-UCH-L1–3CS mutant was also present in the membrane fraction, suggesting that the cysteine modification at Cys-90, Cys-152, or Cys-220 is not involved in membrane partitioning.

Because UCH-L1 has a high affinity for ubiquitin, we tested whether membrane association of UCH-L1 might arise from binding to a protein containing a domain resembling a ubiquitin fold. The Asp-30 site on UCH-L1 is required for ubiquitin binding (22). The D30K mutation abolished UbVME substrate binding (Fig. 3*C*), although the overexpressed HA-UCH-L1-D30K mutant still partitioned into the membrane fraction (∼22%) (Fig. 3*D*). Therefore, together with the data from the 3CS mutant, we conclude that membrane association of UCH-L1 is not dependent on an interaction with the ubiquitin-binding region of UCH-L1.

The I93M UCH-L1 point mutation has been linked to a familial strain of Parkinson disease (23). Interestingly, it has been reported that the I93M mutant increases insolubility (24) and decreases ubiquitin hydrolase activity (23), so we tested if it altered the proportion of UCH-L1^M^. Our results indicate that I93M partitions into the membrane and retains sufficient overall structure to bind UbVME to the same extent as WT UCH-L1.

##### Membrane Association of UCH-L1 Is Not Dependent on Modification to the N- or C-terminal Sequence

**FIGURE 4.**
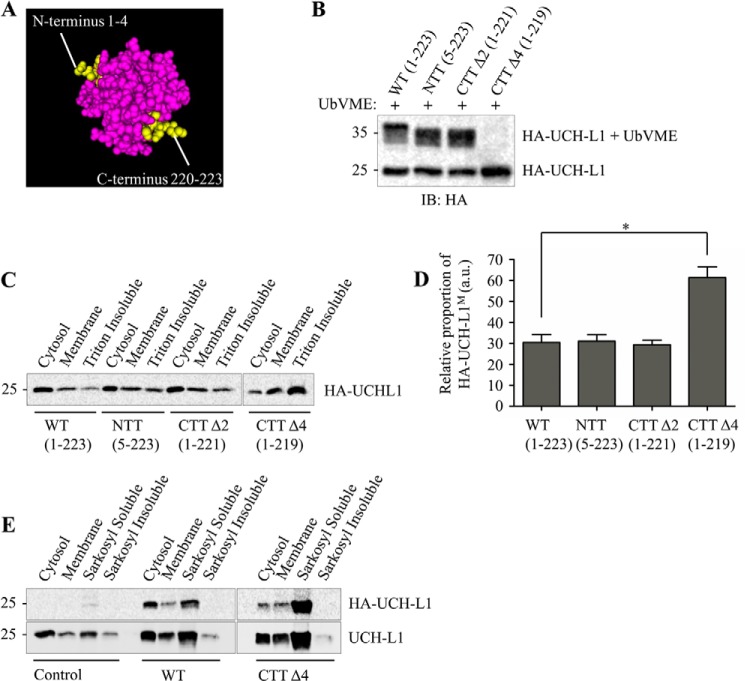
**UCH-L1 C-terminal truncation CTTΔ4 (1–219) leads to misfolding and decreased solubility.** *A*, three-dimensional model showing the location of N- and C-terminal truncations using Cn3D software (38) with UCH-L1 PDB code 2ETL. *B*, deletion of the final four residues of UCH-L1 abolishes UbVME binding. Cultured cortical neurons were infected with Sindbis virus expressing the indicated HA-UCH-L1 constructs. Lysates were incubated with 2 μm UbVME to assess the effect of mutations on substrate binding. *IB*, immunoblot. *C*, deletion of the final four residues of UCH-L1 causes a decrease in protein solubility. Cultured cortical neurons were infected with Sindbis virus expressing the indicated HA-UCH-L1 constructs. The Triton X-100-insoluble pellet was isolated by subjecting the membrane pellet to a further centrifugation step. HA-CTTΔ4-infected cells displayed a loss of soluble UCH-L1^C^ and an increase in signal detected in the Triton X-100-insoluble fraction. *D*, relative proportions of the indicated HA-UCH-L1 constructs detected per microgram of protein in the membrane fraction relative to the cytosolic fraction (*n* = 3). Data were analyzed using one-way analysis of variance with post hoc Bonferroni test. *, *p* < 0.05. *a.u.,* arbitrary units. *E*, Triton X-100-insoluble UCH-L1 can be solubilized using sarkosyl buffer. The 1% Triton X-100-insoluble pellet was resuspended in 1% sarkosyl buffer and subjected to further ultracentrifugation to isolate a sarkosyl-soluble fraction. The final sarkosyl-insoluble pellet was resuspended in 2% SDS. 20 μl of the sarkosyl-soluble and -insoluble fractions were loaded. No HA signal was detected in the sarkosyl-insoluble fractions of HA-WT and HA-CTTΔ4-infected neurons.

To investigate the possible roles of signaling sequences at the N and C termini in UCH-L1 membrane association, we removed the first four N-terminal amino acids (MQLK) and the final two residues (AA) (Fig. 4*A*). UCH-L1 has a complex “knotted” backbone structure, and it has been predicted that even minor N- or C-terminal truncations would denature the protein (25). Our data, however, demonstrate that removing the first four residues or the final two residues from UCH-L1 did not affect its ability to bind UbVME (Fig. 4*B*). Furthermore, there was no significant change to the proportion of HA-UCH-L1 in the membrane fraction with either the N-terminal truncation (5–223) or the C-terminal truncation (CTTΔ2, 1–221) (Fig. 4*C*).

##### C-terminal Deletion of the Final Four Residues of UCH-L1 (CKAA) Leads to Increased Membrane Association and Decreased Solubility

Although the CTTΔ2 truncation behaved the same as the WT, an HA-tagged C-terminal truncation (CTTΔ4, 1–219), which deleted the final four residues (CKAA) encompassing the proposed site of farnesylation, reduced cell viability. Initially we noticed that UbVME binding was abolished (Fig. 4*B*), suggesting that the protein conformation had been altered. Consistent with this, there was a significant loss of the soluble cytosolic form of UCH-L1, with the majority of the signal being detected in a 1% Triton X-100-insoluble fraction. There was also an increase in the proportion of UCH-L1^M^ in neurons infected with the CTTΔ4 mutant compared with the WT (Fig. 4, *C* and *D*).

It has been reported that UCH-L1 is enriched in the insoluble protein aggregates, consistent with several types of neurodegenerative disease (3). To investigate whether the CTTΔ4 mutant is aggregating within the neurons, we further analyzed the Triton X-100-insoluble fraction by resuspending it in a 1% sarkosyl lysis buffer. Although a small proportion of endogenous UCH-L1 remained in the sarkosyl-insoluble fraction, all of the HA-UCH-L1 contained in the Triton X-100-insoluble fraction of both WT and CTTΔ4-infected neurons could be solubilized with sarkosyl (Fig. 4*E*).

##### C-terminal Deletion of the Final Four Residues of UCH-L1 (CKAA) Disrupts the Protein Secondary Structure

**FIGURE 5.**
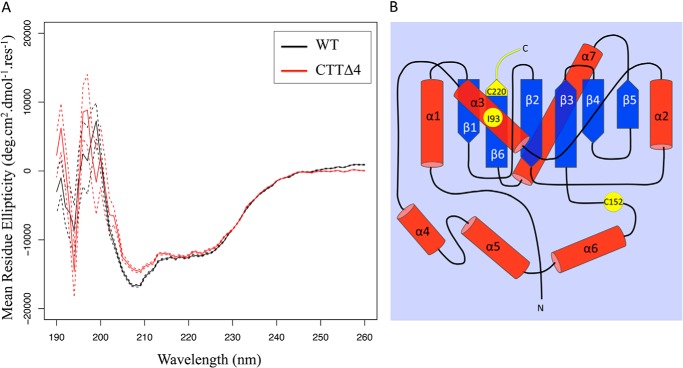
**Analysis of the protein structure reveals increased aggregation of CTTΔ4 compared with the WT.** *A*, circular dichroic spectroscopy data show that the CTTΔ4 mutant (*red line*) has a slightly shallower minimum at 222 nm, indicating that it has a marginally lower α-helical content compared with the WT (*black line*) at 5 μm protein concentration in PBS with 1 mm TCEP and 0.01% SDS. In each case, the protein is approximately one-third α-helical. There is a dramatic increase in the tendency of the CTTΔ4 mutant to aggregate, as shown by the greatly increased scattering evident in the spectrum, especially at lower wavelengths. Data are presented as mean ± S.E. (WT and CTTΔ4, *n* = 64). *B*, topological mapping of residues in UCH-L1 associated with altered protein stability. α-helices are shown in *red*, and β-strands are shown in *blue*. Residues reported to correlate with changes in protein stability are shown in *yellow*.

To explore the possible reasons for the changes in solubility, we analyzed the secondary structure using circular dichroism. The spectra show that the truncated protein has a shallower minimum at 222 nm, indicating a slightly reduced α-helical content compared with the WT (Fig. 5*A*). In each case, the protein is approximately one-third α-helical. The most significant difference between the proteins is the marked increase in aggregation of truncated UCH-L1, as shown by the greatly increased scattering evident in the spectrum, especially at lower wavelengths.

##### C-terminal Deletion of the Final Four Residues of UCH-L1 (CKAA) Leads to Cell Death

**FIGURE 6.**
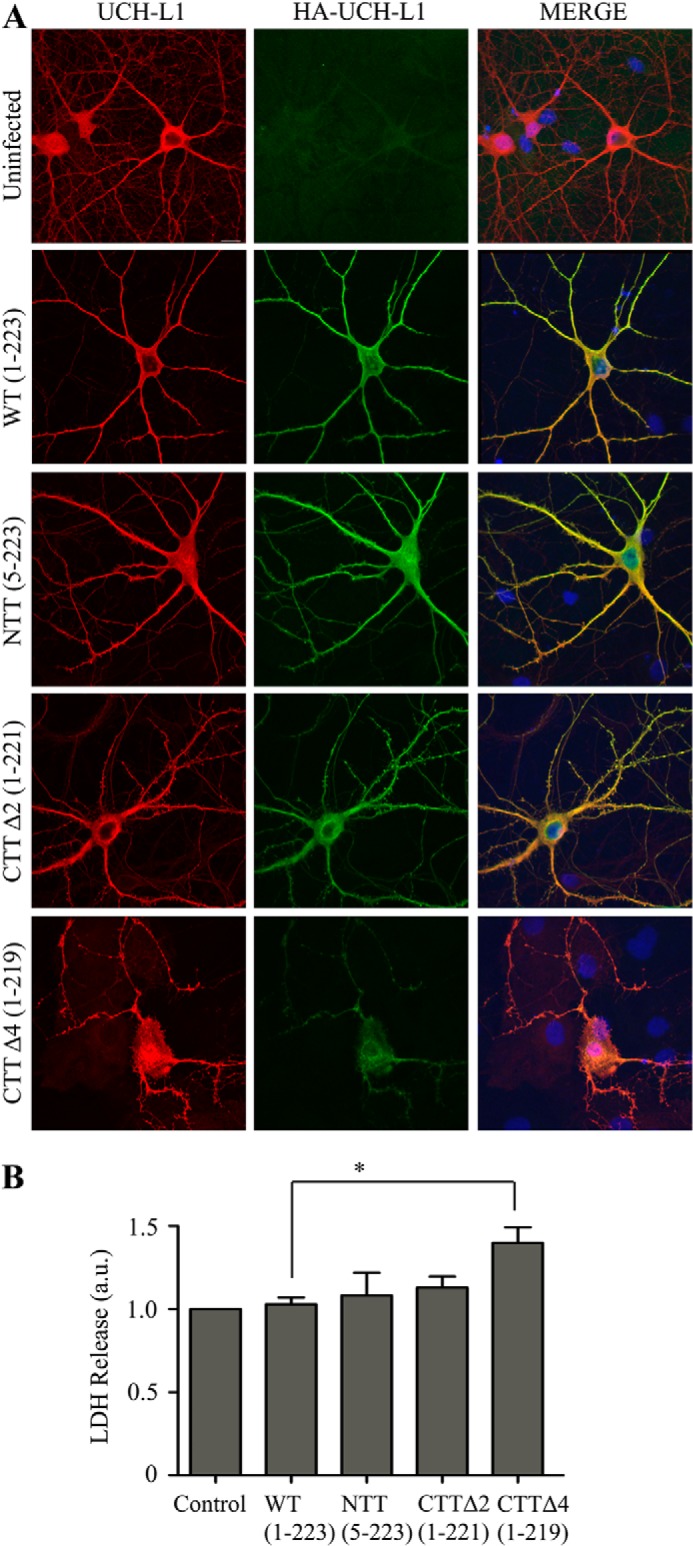
**Deletion of the final four residues of UCH-L1 leads to increased cell death.** *A*, deletion of the final four residues leads to altered UCH-L1 distribution. Hippocampal neurons were infected with Sindbis virus expressing the indicated HA-UCH-L1 constructs to assess the distribution of total (endogenous and infected) UCH-L1 and infected HA-UCH-L1. HA-UCH-L1 distribution in WT, N-terminal truncation, and CTTΔ2-infected cells is comparable with endogenous UCH-L1. Infected protein is largely absent from the processes. *B*, deletion of the final four residues leads to increased cell death. The cell death assay measured the release of lactate dehydrogenase. Infection with Sindbis virus expressing the HA-CTTΔ4 construct caused a significant increase in cell death in cortical neurons compared with HA-WT. *Control*, uninfected. Data were analyzed using one-way analysis of variance with post hoc Bonferroni test. *, *p* < 0.05. Control, HA-WT, and HA-CTTΔ4, *n* = 5; HA-NTT, *n* = 4; HA-CTTΔ2, *n* = 3.

Confocal microscopy of hippocampal neurons infected with the different UCH-L1 constructs using Sindbis virus revealed significant differences in the distributions of CTTΔ4 compared with the WT. CTTΔ4 displayed a non-uniform, mostly perinuclear staining and was largely absent from the processes (Fig. 6*A*). Furthermore, overexpression of this mutant markedly increased neuronal cell death, as measured by lactate dehydrogenase release (Fig. 6*B*).

## DISCUSSION

Given its abundance in neurons, surprisingly little is known about UCH-L1 and especially the mechanisms and consequences of membrane localization (UCH-L1^M^). A previous report proposed that farnesylation mediates membrane association in cell lines (9), but the effects in neurons were not determined. In contrast, in this study, we were unable to detect UCH-L1^M^ by subcellular fractionation in the HEK293T and COS7 cells we tested, although these cell lines both expressed endogenous UCH-L1^C^. Notably, cell lines such as COS7 and HEK293T are derived from tissues that, when healthy, do not express significant levels of UCH-L1. Furthermore, in non-neuronal cells, UCH-L1 has been implicated in the appearance and progression of certain cancers (26), and, therefore, the aberrant expression of UCH-L1 in these cell lines likely arises as a result of the immortalization process.

Our data from primary cortical cultured neurons indicate that ∼30% of UCH-L1 is membrane-associated, and we did detect low levels of UCH-L1^M^ in N2a cells, which are derived from a neuroblastoma, suggesting that membrane association is likely a neuron-specific phenomenon (Fig. 1). The proportion of UCH-L1^M^ was unchanged in differentiated N2a cells following serum starvation, suggesting that, if membrane-association of UCH-L1 is neuron-specific, it is not dependent on neurite outgrowth. The identity of the specific membrane(s) with which UCH-L1 associates has remained unclear because the very high levels of expression of native UCH-L1 in neurons, combined with its relatively uniform distribution, make it difficult to resolve using imaging techniques such as confocal microscopy. Also, unlike the previous report in clonal cell lines (9), partitioning to the membrane in neurons is not dependent on farnesylation because neither the farnesyltransferase inhibitor FTI-276 nor mutation of the Cys-220 residue to serine (Figs. 2 and 3) reduced the proportion of UCH-L1^M^. Furthermore, because farnesylation and geranylgeranylation can only occur at one site at the C terminus, mutation of Cys-220 precludes involvement of any form of prenylation.

We therefore explored other possible causes of membrane association using a range of UCH-L1 mutants. Surprisingly, our results indicate that membrane partitioning of UCH-L1^M^ is not dependent on its affinity for ubiquitin or ubiquitin-like domains, an N-terminal signal sequence, or any C-terminal modification such as glycolipid anchoring. Furthermore, our mutagenesis also ruled out posttranslational modification of Cys-152 or Cys-220, or direct lipid binding via these residues as a route for UCH-L1 membrane association. One possibility is that UCH-L1^M^ is recruited to and retained at the membrane via interacting proteins or as part of a membrane-bound complex. A crystallographic study of UCH-L1 has identified possible protein-protein interaction sites (27), and although no direct interacting proteins for UCH-L1 have been identified, this appears to be a promising avenue for future research.

UCH-L1 is enriched in pathogenic insoluble protein deposits associated with neurodegeneration (28), and an interaction with α-synuclein has been suggested (13). It has been proposed that UCH-L1 possesses E4 ubiquitin ligase activity, which promotes polyubiquitination of α-synuclein to pathologically alter basal turnover (8). Furthermore, it has been proposed that this interaction is mediated specifically by farnesylated UCH-L1^M^ (9), and, on the basis of this finding, small molecule farnesyltransferase inhibitor drugs such as LNK-754 are currently in clinical trials to inhibit or reduce the levels of farnesylated UCH-L1^M^ (29, 30). Although our study does not preclude potential roles for FTIs in treating neurodegeneration, our data strongly suggest that they do not act by decreasing the membrane association of UCH-L1^M^.

UCH-L1 has an unusual knotted backbone structure, and it is believed that extensive N- or C-terminal truncations can destabilize the tertiary structure of the protein (25). In addition, UCH-L1 has been reported to be susceptible to unfolding and aggregation, either through modification or mutation, leading to a gain of toxicity (15, 31). For instance, unfolding may lead to increased association with protein aggregates, such as Lewy bodies (28) and tau tangles observed in Alzheimer disease (14). These events correlate to decreased levels of soluble UCH-L1^C^, but how UCH-L1 participates in these processes is unclear. Interestingly, expression of a UCH-L1 deletion mutant lacking the final four residues (CKAA, CTTΔ4) caused a dramatic increase in neurotoxicity, whereas deletion of the final two residues (AA, CTTΔ2) had no marked effect (Fig. 6). The CTTΔ4 mutant abolished UbVME substrate binding and led to a loss of soluble cytosolic UCH-L1 with a corresponding increase in a Triton X-100-insoluble but 1% sarkosyl-soluble aggregated form. CTTΔ4 showed strong perinuclear staining and a dramatic tendency to aggregate, consistent with a loss of tertiary structure and increased neuronal death.

A common theme that emerges from both point mutation and oxidative modification studies of the misfolding and/or aggregation of UCH-L1 is disruption to the central hydrophobic core (15, 31). Although it has a knotted primary sequence, the tertiary structure of UCH-L1 folds into two α-helical lobes surrounding a tightly packed and solvent-inaccessible hydrophobic core of β-strands (31). Residue Ile-93 is located in α-helix α3, which directly contacts the β6 strand (Fig. 5*B*). The I93M mutation is associated with Parkinson disease and has been reported to decrease UCH-L1 solubility and hydrolytic activity (24, 31). These effects have been attributed to disruption of the β6 strand in particular as well as β2–4 (31), and it has been proposed that this leads to increases in UCH-L1 insolubility (24). However, it should be noted that another study reported that the I93M mutant is well folded and structurally similar to the wild-type protein (15). Overall, our data are most consistent with the latter finding because we detected little change in the solubility of the I93M mutant (Fig. 3*D*).

Cyclopentenone prostaglandins such as 15d-PGJ2 are produced during an immune response or under oxidative stress. 15d-PGJ2 specifically modifies UCH-L1 at C152 site, leading to a loss of protein stability (15). Although C152 is situated in a short unstructured loop covering the active site of the protein, it was proposed that 15d-PGJ2 acts as a lipophilic wedge to penetrate into the core of the protein, disrupting the tightly packed structure of the protein (Fig. 5*B*). Analogous to this disruption of the packed core structure by prostaglandins, our results indicate that disruption of the extreme C terminus may also have a similar effect and provide a potential mechanism for how UCH-L1 structure and stability may be regulated by other forms of oxidative stress.

Crystal structures of UCH-L1 suggest that Lys-221 might be integral to the tertiary structure by forming intramolecular bonds to hold the three-dimensional shape of the protein (PDB code 2ETL, Ref. 27). Removal of the final four residues, 220–223, from UCH-L1 would remove the intramolecular hydrogen bonds formed by the Lys-221 residue and would also expose the protected β6 strand to the solvent, causing a sufficient disturbance to the hydrophobic core that the protein loses significant tertiary structure without necessarily losing the significant localized α-helical secondary structure. This phenomenon occurs in the misfolding of other proteins, resulting in the exposure of flexible, previously hidden hydrophobic regions that mediate aberrant interactions with proteins and cellular membranes. Indeed, these kinds of conformational transitions lead to the assembly of protein oligomers characteristic of many neurodegenerative diseases, including amyloid-β in Alzheimer disease and α-synuclein in Parkinson disease (32, 33). The resultant aggregates disrupt essential cellular processes by forming membrane pores (34), disrupting membrane cholesterol (35), interfering with protein chaperone machinery (36), and/or sequestering essential proteins to the aggregates (37).

We therefore propose that exposure of the hydrophobic core causes a toxic gain of function in which misfolded UCH-L1 aberrantly binds to hydrophobic intracellular membranes, and this leads to cell death. The CTTΔ4 mutant we report here represents an extreme form of unfolded UCH-L1. Importantly, we attribute this to a toxic gain of function rather than a loss of hydrolytic activity because C90S and D30K do not have the same effect. Taken together, our findings suggest that modifications or mutations that alter the folding and/or stability of UCH-L1 are neurotoxic. The CTTΔ4 truncation provides a useful new tool for studying how UCH-L1 misfolding and dysfunction lead to protein instability and cell death, which pathways are involved, and what measures can be taken to reduce or prevent toxicity.

In conclusion, our results demonstrate that membrane association of UCH-L1 is differentially regulated in different cell types. We investigated a series of possible mechanisms for the membrane association, and our data indicate that, contrary to a previous report, farnesylation and/or other cysteine posttranslational modifications are not responsible for membrane partitioning. Therefore, further work is required to define how UCH-L1 is recruited and retained at membranes selectively in neurons and to elucidate the functional consequences of this membrane association.

## Footnotes

2 The abbreviations used are:

UbVME: ubiquitin vinyl methyl ester
3CS: three cysteines
CTT: C-terminal truncation mutant
FTI: farnesyl transferase inhibitor.
